# State of the Art and Future Perspectives of Atmospheric Chemical Sensing Using Unmanned Aerial Vehicles: A Bibliometric Analysis

**DOI:** 10.3390/s23208384

**Published:** 2023-10-11

**Authors:** Diego Bedin Marin, Valentina Becciolini, Lucas Santos Santana, Giuseppe Rossi, Matteo Barbari

**Affiliations:** 1 Department of Agriculture, Food, Environment and Forestry, University of Florence, Via San Bonaventura, 13, 50145 Florence, Italy; diego.marin@ufv.br (D.B.M.); giuseppe.rossi@unifi.it (G.R.); matteo.barbari@unifi.it (M.B.); 2 Department of Environmental Engineering, Federal University of Lavras, Aquenta Sol Avenue, P.O. Box 3037, Lavras 37200-900, Brazil; lucas.unemat@hotmail.it

**Keywords:** drones, air pollutants, sensors, greenhouse gases, particulate matter

## Abstract

In recent years, unmanned aerial vehicles (UAVs) have been increasingly used to monitor and assess air quality. The interest in the application of UAVs in monitoring air pollutants and greenhouse gases is evidenced by the recent emergence of sensors with the most diverse specifications designed for UAVs or even UAVs designed with integrated sensors. The objective of this study was to conduct a comprehensive review based on bibliometrics to identify dynamics and possible trends in scientific production on UAV-based sensors to monitor air quality. A bibliometric analysis was carried out in the VOSViewer software (version 1.6.17) from the Scopus and Web of Science reference databases in the period between 2012 and 2022. The main countries, journals, scientific organizations, researchers and co-citation networks with greater relevance for the study area were highlighted. The literature, in general, has grown rapidly and has attracted enormous attention in the last 5 years, as indicated by the increase in articles after 2017. It was possible to notice the rapid development of sensors, resulting in smaller and lighter devices, with greater sensitivity and capacity for remote work. Overall, this analysis summarizes the evolution of UAV-based sensors and their applications, providing valuable information to researchers and developers of UAV-based sensors to monitor air pollutants.

## 1. Introduction

Anthropogenic pollution is the result of the introduction of substances that induce physical, chemical or biological changes in natural resources into the environment and that cause detriment to living organisms or ecosystems. The assessment of air pollutants emissions has long been enforced through atmospheric chemical sensing to achieve air quality standards, both from the perspective of meeting environmental sustainability and human health goals. Multiple chemical species are involved in air emission processes, comprising both air pollutants and greenhouse gases.

Among long-term monitored air pollutants are volatile organic compounds (VOCs), particulate matter (PM), NOx, O_3_, SO_2_ and CO [1]. Air pollutants are mainly absorbed by humans through breathing and contribute to developing diseases of the respiratory system, as well as reproductive disorders and allergies [2]. In addition, air pollution decreases visibility, sunlight and crop yields and causes ambient air to warm [3,4]. Threats caused by air pollution have a wide range of impacts—they concern a spatial scale from the local to global scales and a time scale from hours or days to many years [5]. Therefore, the precise and continuous monitoring of atmospheric pollutants is essential to guarantee the health of human beings and ideal conditions for the environment.

As for greenhouse gases (GHGs), the increase in the atmospheric concentration of anthropogenic GHGs, such as CO_2_, CH_4_, N_2_O and SO_2_ determines Earth’s surface warming at a global scale, thus affecting climate and, as a consequence, the stability of ecosystems and their productivity. Locating and quantifying sources of GHGs, thus, is crucial to meeting the goal of climate neutrality.

Air quality is traditionally measured with stationary monitors using standard air pollution monitoring devices located at representative sites. These devices are highly consistent, accurate and capable of measuring a wide range of pollutants. The disadvantages of these monitoring tools are their large size, weight and cost [6]. These tools also lack spatial characteristics, for example in the context of analyses of concentration variability, along with height [5]. Unmonitored locations are then supported through modelling by using interpolation methods.

As an alternative to traditional methods, unmanned aerial vehicles (UAVs) have been increasingly used to monitor and assess air pollutants and GHGs. UAVs cover large areas and can continuously monitor remote, dangerous or difficult-to-access locations, increasing the operational flexibility and the resolution over land-based methods [7]. Interest in the application of UAVs for monitoring air pollutants is also evidenced by the recent emergence of sensors specifically designed for UAVs or even UAVs designed with integrated sensors [8]. Currently, it is possible to find sensors with the most diverse specifications, types of measurement, precision, weight and energy consumption. Therefore, the choice of ideal sensor will depend on the type of study, the available budget and the load capacity of the used UAV.

Faced with the amount of information emerging from sensors for monitoring air pollutants and GHGs in the literature, the application of a bibliometric analysis to investigate this evolution makes it possible to map the theme dynamically, highlighting the agents involved in its scientific production (research, researchers and institutions), demonstrating the trends and association with regions of occurrence. Bibliometric analyses use quantitative and qualitative measures to assess scientific impacts through metrics such as citation counts, author production lists, national or thematic bibliographies, and publication patterns [9]. Systematic reviews aim to answer a specific research question through a methodological strategy involving synthesizing the literature on the subject [10]. 

Given the relevance of studies on UAV applications for monitoring pollutants, this research aimed to present the main scientific contributions on this topic in the recent decade, between 2012 and 2022. Through bibliometric mapping, this work explores the trends, technologies and applications of UAV-based atmospheric chemical sensing and presents the evolution of research over the years, the most cited articles, the most relevant journals, the main countries, the trends and the innovations.

## 2. Materials and Methods

Bibliometrics uses mathematical and statistical methods to analyse and measure the metadata of publications, to organize and analyse information and to examine bibliographic material [11]. This approach allows for identifying dynamics and possible trends in scientific production [12] by exposing the trajectory of traditional and emerging publications to outline future investigations. 

Although bibliometrics provides new insights supported by the objective quantitative strength of the methodology [13], the method cannot be considered an alternative to traditional theoretical literature reviews. They are complementary, as they help the researcher to choose the most important studies in the area, the most influential researchers of the subject, the basic topics in the field, as well as the research institutions and countries [14]. 

The sequence of work adopted in this bibliometric analysis was divided into data retrieval, pre-processing, network extraction, normalization, mapping and visualization analysis [15]. Following the methodology proposed by [16,17], we conducted a comprehensive search of journal articles to cover the entire research domain for the application of UAV-embedded sensors for the monitoring and assessment of air pollutants and greenhouse gases:

1. In the first step, we chose the most comprehensive and reliable databases with standardized results related to all applications of sensors embedded in chemical sensing UAVs. We then analysed selected articles, removing all research outside the topic of analysis.
2. The evolution of the number of articles published in the selected period was analysed to highlight the expansion and trend in publication output in the literature.
3. We analysed and explored the most cited articles to identify the most impactful research in the field.
4. The most influential countries and journals and the importance of these countries in the study subject were assessed.
5. A keyword analysis was elaborated upon to investigate and analyse the most important and trending topics keywords in the search area.
6. Finally, we disclosed the research trend and future direction in air sensing by UAV.

### 2.1. Selection of Research Databases

The databases selected for carrying out the research were Scopus and Web of Science due to their relevance in bibliometric studies and the reliability of the data. In addition, they represent the most consulted databases worldwide. Although some studies tend to use only one of the databases due to the technical complexity of data combination, we chose to use both databases together. Studies show that these two databases only overlap by 49–50% [18]. Therefore, research on different scientific bases is essential for correctly interpreting and using bibliometric indicators in evaluating scientific research [19].

### 2.2. Data Research Criteria

In this study, we chose to select only publications of peer-reviewed scientific articles in English published between 1 January 2012 and 31 December 2022. Search terms were defined before querying the selected databases. Different searches were carried out to define the keywords to identify the ones most used by authors in the field of study. The research was based on a combination of the following keywords in the titles, abstracts or keywords (and their acronyms): (UAV* OR RPA* OR “unmanned aerial vehicle” OR “drone” OR “fixed wing” OR “rotary wing”) AND (“gas sensor” OR “gas sensing” OR “chemical sensor” OR “chemical sensing” OR “particulate matter sensor” OR “greenhouse gas sensor” OR “optical gas sensor” OR “electronic nose” OR “amperometric gas sensor” OR “photoionization detector” OR “non-dispersive infrared sensor” OR “MOS sensor” OR “metal oxide chemirisistive sensor” OR “laser absorption spectroscopy” OR “cavity-enhanced absorption spectroscopy” OR “off-axis integrated cavity output spectroscopy” OR “tunable laser diode” OR “cavity ring-down spectroscopy” OR “optical particle counter” OR “thermal infrared” OR “multi-sensor system” OR “air quality” OR “air pollution” OR “gas emission”).

### 2.3. Selection and Organization Procedures

The selection and organization process consisted of two stages. In the first step, we removed duplicate articles, as the Scopus and Web of Science research databases can present the same article. In the second stage, conceptual works without applications were removed. This study focused only on articles that demonstrated flight tests in real conditions and presented a detailed description of the UAVs and the sensors used for monitoring air pollutants. The selection and organization process resulted in 136 documents. Data were organized in an electronic spreadsheet and imported into a bibliographic analysis software.

### 2.4. Bibliometric Analysis

Bibliometric mapping was performed using VOSviewer (version 1.6.17); this software offers a range of intuitive visualization, particularly for analysing bibliometric maps [20]. VOSviewer is used to build networks of items: researchers, scientific journals, countries, keywords and publications connected through links based on co-authorship, co-occurrence, citation, bibliographic coupling or co-citations [11]. Based on a multidimensional mapping technique, VOSviewer locates words in a dimensional space, portraying the distance between items according to their similarity [16]. The results are presented in a circle, representing items found in the search. These items are grouped and represented by colour, forming a bibliometric map [21]. Quality criteria for research and journals are citations and scientific impact, as reported by [22]. This rule was used for bibliometric mapping, which took into account the annual evolution of publications and citations, the most influential countries in publications related to this field, the most notable journals, the main keywords used by authors, the main keywords found in the most important publications, and the trends and terms that indicate future lines of research.

## 3. Results and Discussion

### 3.1. Temporal Evolution of Publications

**Figure 1:**
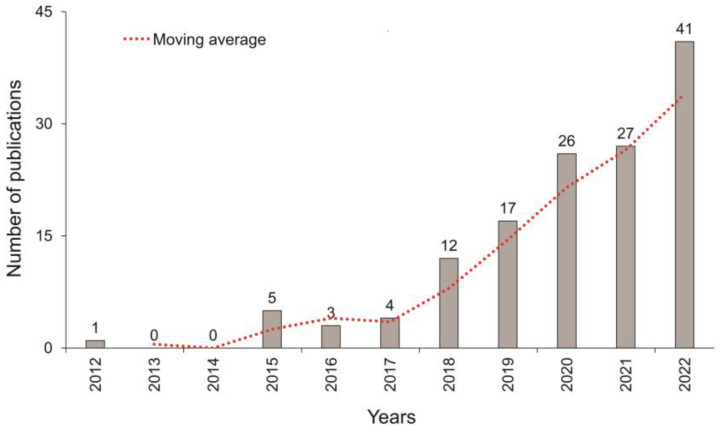
Temporal distribution of research publications concerning UAV-based sensors for atmospheric chemical sensing from 2012 to 2022.

The bibliometric analysis found 136 scientific papers on UAV-based sensors for air sensing from 2012 to 2022. The temporal distribution of these publications is shown in Figure 1, illustrating the publications for each year. From 2012 to 2017, few studies used sensors embedded in UAVs to monitor air pollutants or greenhouse gases. Between the years 2012 and 2015, most studies evaluated the vertical distribution of PM_2.5_ and PM_10_ in open spaces. For this, they mostly used fixed-wing UAVs, developed or adapted by the authors, equipped with sensors based on lasers. In the following years, 2016 and 2017, laser-based sensors continued to be used in various applications, such as highway monitoring and characterization of plumes emitted by burning military ammunition. However, those studies have mostly used multirotor aircrafts, taking advantage of recent improvements in consumer UAV technology and their abilities to manoeuvre in the horizontal and vertical dimensions, maintaining a fixed position in the air even under high wind conditions [23]. In addition, the need to develop low-cost sensors began.

As of 2018, there has been a significant increase in studies driven by the development of new technologies for consumer UAVs equipped with low-cost mobile commercial microsensors. These sensors offer numerous advantages for capturing air pollutants’ spatial and temporal variabilities and for providing measurements with high sensitivities and fast response times (minutes to seconds) [24]. During the period 2018 to 2022, most studies focused on air pollutants detection in urban areas (38 studies); open fields (22 studies); highways (13 studies); waste and wastewater sites (12 studies); and fossil fuel mining, storage and transport (10 studies). However, there were also studies evaluating air pollutants in industrial areas, volcano plumes, forest fires, agriculture and ships.

#### Platforms and Sensing Technologies for UAV-Based Chemical Sensing

The analysis of the 136 papers highlighted the state of the art of sensing technologies and types of UAVs suitable for atmospheric chemical sensing. Two main types of platforms were employed in the selected literature: fixed-wing (FW) and rotary-wing (RW) UAVs. FW drones were employed as sensing platforms in 16 papers, with two types of structures: model aircraft (8 papers) and lightweight fixed-wing UAVs (8 papers). FW UAVs can fly at high speeds, covering large areas in one flight. The primary limitation is the lower spatial resolution compared with RW platforms, which may be influenced by the response time of the onboard sensors. Further, they require a launch system or a take-off and landing runway [5,8]. In lightweight fixed-wing UAVs, payload capacity is low and affects the total weight of the sensing equipment that can be mounted on the platform. Accordingly, in the literature selected for this analysis, the sensing payload weight ranged from 0.3 and 0.92 kg [25,26,27,28]. In research involving model aircraft, the payload weight ranged from 0.54 to 3.1 kg [29,30,31,32,33,34]. RW platforms were the preferred type of UAV for air quality monitoring, being employed in 118 papers. Their capacity for vertical take-off and landing, high manoeuvrability, slow flight speed and high payload capacity determined their success in this research field, with growing contributions since 2018. 

Based on the applied research studies compiled in our analysis, the sensing technologies available for UAV-based air quality monitoring allowed for the detection and measurement of a wide array of air pollutants: CO, CO_2_, CH_4_, PM, VOC, NO, NO_2_, NH_3_, O_3_, SO_2_, H_2_S and black carbon (BC) were the prevalent target substances. The required instrumentation can vary depending on the sensing principle, cost, weight, accuracy and target pollutant. Low-cost sensors available on the market, with a price lower than USD 1000, were widely used in this field in the past 10 years. In this category, four types of commonly used sensors were included: electrochemical sensors (EC), metal oxide semiconductor (MOX or MOS) sensors, non-dispersive infrared (NDIR) sensors and light-scattering laser photometers (LS) [8]. In 41 papers, EC sensors were successfully mounted on UAVs to measure the concentration of the abovementioned list of gases, with the exception of CH_4_, PM, VOC and BC. MOX sensors were tested in 11 studies related to in-flight monitoring of CO, CH_4_, VOC, NO_2_, NH_3_, O_3_, and H_2_S. NDIR sensors are miniaturized optical analysers based on the property of certain molecules to absorb light at specific wavelengths. They are considered well suited for accurately determining CO_2_ concentrations since the molecule has a non-overlapping absorption peak [8]. NDIR sensors were applied for UAV-based sensing of CO_2_ in 21 papers. The light-scattering (LS) technique consists of a light source illuminating an air channel where a particle deviates the light beam based on the principle of light scattering. Optical sensors based on light scattering are considered optimal candidates for miniaturized systems, given their sensitivity and simplicity [35]. Forty-six studies applied LS sensors to UAVs for the assessment of particulate matter (PM_1_ to PM_10_). 

To monitor several pollutants at the same time, some studies combined different types of low-cost sensors simultaneously, usually up to 10 sensors for particulate or gaseous species. These multi-sensor systems host multiple sensors and contain all the necessary electronics, gas transmission paths, data acquisition and power management systems [5]. Recently, studies based on electronic nose sensors (e-nose) have been carried out. E-noses are devices that mimic the functions of the human nose through a series of combined gas sensors, usually MOX sensors, using machine learning algorithms [36].

However, for some applications, i.e., the determination of CH_4_ concentration, these sensors are not able to guarantee the accuracy and data quality of high-accuracy optical gas analysers. These analysers produce absorption spectra of the analysed molecules when they are irradiated with infrared or ultraviolet light [5]. To increase the quality of measurements, high-accuracy optical instruments include sophisticated components such as lasers, high-reflectivity mirrors, quartz-coated cavities, and temperature and pressure compensation systems, which result in bulky, heavy, power-hungry and expensive instruments [8]. The most frequently applied laser absorption (LAS) technique, i.e., TDLAS, is based on a tuneable laser diode (TDL). The measurement can be performed with the laser beam operating in closed-path (CP-TDL) designs or via open-path (OP-TDL) designs [37]. Modifications of the TDLAS technology led to the development of new methodologies, such as the stand-off or back-scattered TDLAS (sTDLAS). Compared with traditional OP-TDL systems, the sTDLAS detector measures the cumulative gas concentration across the entire beam, collecting information over a large area with a single measurement [5]. Lightweight TDLAS sensors optimized for use on UAVs (weight: 0.53–2 kg) were tested in six studies [38,39,40,41,42,43] to measure CH_4_.

Another group of LAS techniques is cavity-enhanced absorption spectroscopy (CEAS), which uses a resonant optical cavity where laser light bounces back and forth (about 100,000 times), achieving very long effective optical paths of several kilometres [44]. Two main techniques of CEAS are cavity ring-down spectroscopy (CRDS) and off-axis integrated cavity output spectroscopy (OA-ICOS) [5]. According to our analysis, only one study applied a 4 kg CRDS instrument to UAV-based sensing [45].

Several studies were also carried out by applying optical particle counters and thermal cameras. Optical particle counters are enormous and quite expensive instruments. They only process one PM size at a time (e.g., PM_2.5_), cannot perform real-time measurements and cannot output particle number counts [5]. Thermal cameras are useful for leak detection but not for flow quantification without additional support measurements [37]. It is worth mentioning that some sensors can be affected by the downwash of a rotary-wing aircraft. To overcome this limitation, some studies used tubes connected to the aircraft and high-precision sensors on the ground. However, the main disadvantage of this method is that the application of long tubes can cause an increase in the response time due to the adsorption of certain gases on the inner walls of the tubes [5].

### 3.2. The Top 10 Most Cited Articles and Their Relevant Characteristics

A list of the most cited articles is reported in Table 1. Among the 136 articles analysed, 10 had more than 50 citations, and the top 3 most cited had more than 100 citations.

In [24], the best position to attach CO_2_, CO, NO_2_ and NO sensors to an S800 EVO hexacopter manufactured by DJI (Shenzhen, China) was established to measure the emissions of a diesel engine. The gas-sensing payload included three Alphasense gas sensors (Alphasense, B4 type, Great Notley, Essex, UK) and one SprintIR CO_2_ sensor. The Alphasense sensors were electrochemical cells used to measure CO, NO and NO_2_ that operate in amperometric mode and generate a current that is linearly proportional to the fractional volume of the measured gas. The SprintIR sensor measured CO_2_ based on non-dispersive infrared (NDIR) technology. The results of this study demonstrated that the best mounting point is along the X-axis with a distance beyond the helices between 1000 and 1200 mm.

The authors of [38] developed sensors to measure greenhouse gases (CO_2_, H_2_O and CH_4_) designed specifically for unmanned aerial vehicle (UAV) platforms and tested the sensors in a robotic helicopter UT-Dallas Align T-REX 700E. The key innovations were coupling very low-power vertical cavity surface emitting lasers (VCSELs) with low-power drive electronics and sensitive multi-harmonic wavelength modulation spectroscopic techniques. Each sensor consumes less than 2 W of electrical energy with an overall mass between 1 and 2 kg, including batteries. In initial field tests, the sensors flew successfully aboard a T-Rex Align 700E robotic helicopter and showed an accuracy of 1% or less for all gases.

In Ref. [46], the development of a UAV (based on the Green Falcon developed at QUT and the Australian Research Center for Aerospace and Automation) powered by solar energy and equipped with a commercial off-the-shelf NDIR sensor (CDM30K, Figaro Inc., Osaka, Japan) to measure CO_2_ concentrations is presented. This sensor is factory pre-calibrated at 0 and 400 ppm. The accuracy of the sensor was tested against an LI-840, a carbon dioxide analyser, resulting in a measurement error of 5%. Field results demonstrated the ability of the UAVs to capture, analyse and geolocate a gas sample during flight operations. 

Ref. [47] presented the design and characterization of an embedded platform for mapping applications and locating gas distribution leaks using UAVs as mobile operators and CO and VOC metal oxide sensors (MiCS-5121, MiCS-55225; SGX Sensortech Ltd., Neuchâtel, Switzerland). The platform was tested over university facilities to evaluate the sensitivity of MOX sensors. The results showed that the analysis of the target environmental parameters is not disturbed by the airflow generated by the propellers and that the developed system is sensitive to the presence of gases.

To study the vertical distribution pattern of PM_2.5_ concentrations close to the ground (below 1000 m height), the authors of [25] performed a study based on a Sidepak AM510 PM sensor array attached to a light fixed-wing UAV to collect data at 300 m–1000 m heights. The sensor is a robust laser photometer capable of recording PM mass concentrations of up to 20 mg m^−3^, for 1 s intervals, in real time. The study demonstrated the feasibility of UAVs with mobile monitoring devices as an effective and flexible means to collect three-dimensional data on the vertical profile of PM_2.5_.

The research published in [48] used two optoelectrical dust sensors, GP2Y10 (SHARP, Sakai, Japan) and DSM501A (Samyoung, Seongnam, South Korea), installed in two different UAVs (on fixed and rotary wings) to monitor PM10 after blasting at open-pit mine sites. The GP2Y10 sensor utilizes a sharp optics-detecting framework that identifies mirrored light of dust with an IR optics sensor. Measurements were made in a range of 0 to 0.8 mg m^−3^. The authors reported the feasibility of the sensor attached to UAVs to measure PM_10_; however, they also emphasized the need for further investigations and studies in sensor selection and calibration, as well as flight planning.

In [49], a lightweight remote-controlled whole air sampling component (WASC) was designed. They coupled it to an octo-rotor multicopter UAV (Spreading Wings S-1000, DJI Innovations, Shenzhen, China) to perform exhaust sampling of CH_4_, CO_2_, CO, and VOCs in tunnel roads. However, the authors of this study did not attach the sensor for the gas analysis to the UAV. Air samples were collected by WASC and analysed on land with a high-precision terrestrial cavity ring-down spectroscopy (CRDS) sensor (G2401, Picarro Inc., Santa Clara, CA, USA). Although the results were satisfactory, future studies that integrate more devices or sensors in a WASC multicopter for simultaneous sampling and observation of other airborne species are being planned by the authors.

Ref. [50] explored the usefulness of a quadrotor unmanned aircraft system (UAS) as a sampling platform to measure vertical and horizontal CO_2_ concentration gradients at high spatial resolution (1 m) within the mixed layer (0–100 m). An autonomous 3D Robotics Iris+ UAS quadcopter was equipped with a commercial model K-30 CO_2_ sensor. The K30 is a low-cost, low-power CO_2_ meter that uses non-dispersive infrared (NDIR) waveguide technology and an automatic background calibration algorithm to detect CO_2_ between 0 and 10,000 ppm. The K-30 has a precision of ±20 ppm and ±1% of the measured value, with an accuracy of ±30 ppm and ±3% of the measured value within specifications. The proposed system demonstrated high accuracy in the vertical (±0.5 m) and horizontal (±1 m) positions.

A remote-controlled aircraft, model AMR Payload Master 100, was equipped with a custom laser-based methane sensor by [29] to quantify the methane leak rate and its variability in a compressor station in the Barnett Shale. The open-path laser-based sensor provides fast (10 Hz) and precise (0.1 ppmv) measurements of methane in a compact package, while the remote-controlled aircraft provides nimble and safe operation around a local source. More generally, the results demonstrate the unique advantages and challenges of platforms such as small unmanned aerial vehicles to quantify local emission sources to the atmosphere.

A prototype for monitoring air pollutants was designed and developed in [51]. The prototype consists of three main components: a UAV mounted on the frame of an S550 hexacopter, two atmospheric pollutant sensors and a data fusion module. The sensors used were a particulate matter sensor (OPC-N2, Alphasense Inc., Essex, UK) and a NO_2_ sensor (NO_2_-B43F, Alphasense Inc., Essex, UK). The particulate sensor counts particles optically and converts numerical concentrations into mass concentrations of PM_1_, PM_2.5_ and PM_10_, while the NO_2_ sensor uses four electrodes that produce current signals proportional to the gas concentration. The results confirmed the viability of the prototype, demonstrating that it has a stable and high-precision spatial–temporal platform for collecting air samples.

### 3.3. Most Influential Countries and Journals

**Figure 2:**
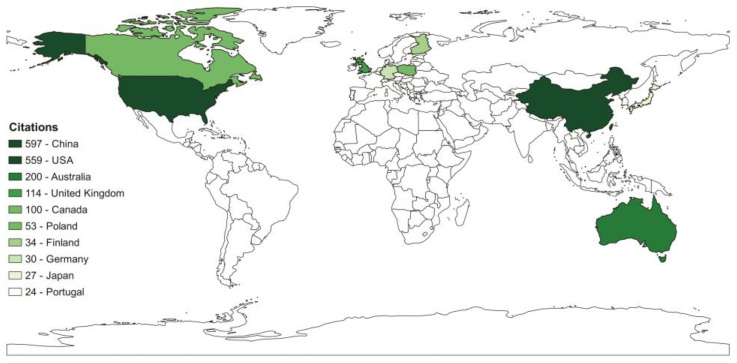
Total citations by country from 2012 to 2022 in the Scopus previewer and Web of Science databases.

The map representing the most influential countries concerning studies developed in this research field is reported in Figure 2. Through this analysis, we seek to better understand the geographic distribution of scholars who contribute most to the application of sensors onboard UAVs in monitoring and evaluating air pollutants. From a country point of view, China, the USA and Australia top the list regarding the number of citations. 

Due to rapid economic development, China faces significant public health challenges, such as declining air quality caused by increased resource use and energy consumption [52,53]. In addition, meteorological disasters such as frequent sandstorms in East Asia are also attributed to air pollution. The United States of America, the second country with the highest number of publications, and China have serious air quality problems mainly due to dependence on fossil fuels, especially in transport. On the other hand, Australia faces serious air quality problems driven by recurrent forest fires that affect not only the air quality in its region but also that in other countries such as New Zealand, Argentina and Chile.

All these reasons above justify China, the USA and Australia being the countries with the most research on UAV-based sensors to monitor air pollutants; however, other factors must be considered. For example, China and the USA are world leaders in the market and in technologies for UAVs and sensors for measuring atmospheric pollutants. In addition, both global and local pressures on the governments of these countries to reduce pollutant emissions have been successful. Australia recently passed some of the most radical environmental legislation in the world to combat climate change. The resolution, approved with 89 votes in favour and 55 against, pledges to reduce harmful gas emissions by 43% compared with 2005 levels by 2030, that is, in just eight years. The US enacted the “Clean Air Act” in 1970, which underwent major amendments in the following years [54], and that reduced emissions of particulate matter, sulphur oxides, nitrogen oxides, carbon monoxide, volatile organic compounds and lead by 73% [55]. China, after experiencing its highest levels of pollution and facing public awareness and criticism, has implemented an increasing number of novel national air control regulations since 2013. Among them are the Environmental Protection Law, the Law on the Prevention and Control of Atmospheric Pollution, and the Environmental Protection Tax Law [56]. The policies produced a severe reduction in PM pollution from 2013 to 2020, with particulate levels decreasing by 39.6%. Among the international policies, the Kyoto Protocol and the Paris Climate Agreement are two key international legal instruments for the regulation of GHG emissions.

Our research included a wide variety of journals, covering virtually all research related to the study topic (Table 2). The journal *Sensors* had the highest number of articles (22) and citations (446), followed by *Atmospheric Environment* with 10 articles and 223 citations and *Atmosphere* with 18 articles and 186 citations. As expected, the journal *Sensors* came out on top of the list, providing an advanced forum for sensor science and technology and its applications. The journals *Atmospheric Environment* and *Atmosphere* publish scientific articles with atmospheric relevance to emissions and the deposition of gaseous and particulate compounds; chemical processes and physical effects on the atmosphere; as well as the impacts of changing atmospheric composition on human health, air quality, climate change and ecosystems.

**Figure 3:**
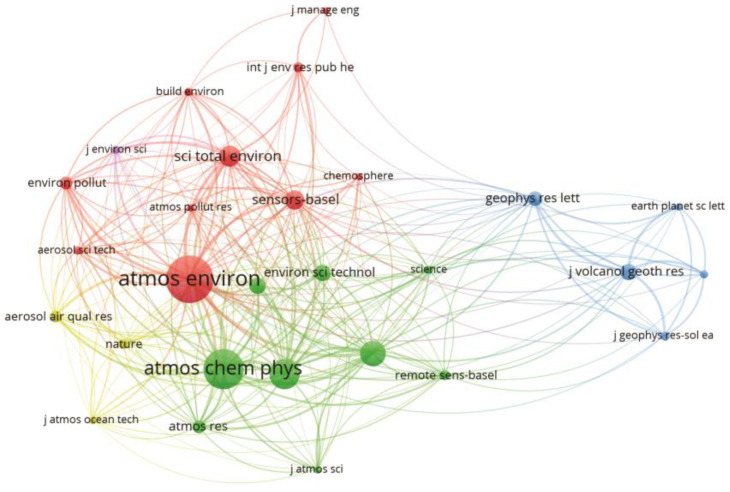
Scientific mapping of the co-citation of journals.

The relationships between scientific organizations that produce knowledge about the study area are shown in Figure 3. The co-citation map of journals is of fundamental importance in bibliometric analysis as it establishes trends and relationships between these organizations.

The co-citation analysis produced three large clusters. The red and green clusters consist of journals such as *Atmospheric Environment*, *Atmospheric Chemistry and Physics*, and *Science of the Total Environment*, which are journals that investigate the Earth’s atmosphere and the underlying chemical and physical processes, while the journals *Sensors* and *Remote Sensing* produce research in the area of remote sensing, which includes the development of sensors and UAVs and applications in several areas of study, including air quality. The blue cluster consists of journals such as *Geophysical Research Letters* and *Journal of Volcanology and Geothermal Research*, which publish papers on significant advances that span all major geoscience disciplines.

### 3.4. Keyword Mapping

One way to investigate the field of study is to analyse the authors’ keywords with the highest occurrence rates in all documents. The keywords that authors select for a publication affect how the article is represented and communicated within scientific communities [17]. Keyword analysis, a tool to reveal broader research trends and directions, refers to compiling the keywords of all related publications in a domain [57]. 

**Figure 4:**
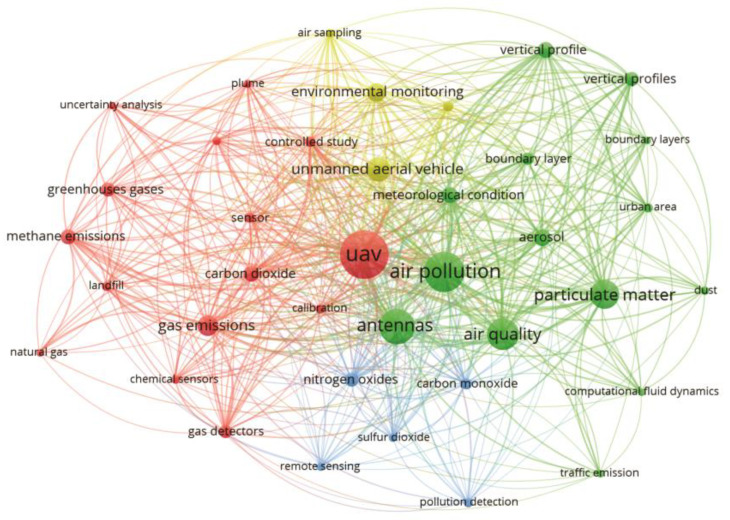
Map of network among author’s keywords. Lines indicate co-occurrences between terms.

As observed in Figure 4, among the 1909 keywords identified in the studies, the term “UAV” appeared most frequently, with 129 occurrences, followed by the terms “air pollution” (95 occurrences), “antennas” (78 occurrences), “air quality” (65 occurrences), “particulate matter” (56 occurrences) and “gas emissions” (34 occurrences). Although our study aimed to explore the application of different types and/or technologies on board UAVs for monitoring and evaluating air pollutants and GHGs, the frequency of terms related to sensor type or technology was not expressive. This occurred because the authors of the study area focused on broader terms, or more generic terms, when describing the title, abstract and keywords of their studies, to the detriment of the type or technology of used sensor.

In Figure 4, two main distinct groups can be identified. The red group associates UAVs with the emission of gases, while the green group associates air pollution with atmospheric conditions. This indicates that the work in this area of study focused on using UAVs to analyse the dispersion of pollutants in the atmosphere, both those affecting primarily human health and those having an impact on the environment. Publications dealing with UAV-based monitoring of gaseous compounds recognize the importance of sensor calibration to improve the accuracy of results, as attested to by the recurrence of the keyword “calibration”. This procedure is common, particularly when working with low-cost sensors or with electrochemical sensors where the response is well affected by changes in environmental conditions such as temperature [58]. Calibration is achieved, e.g., by adjusting the response of sensors previously calibrated in laboratory conditions by manufacturers using parallel measurements with reference equipment, to ensure optimal work under field conditions. Studies focusing on environmental sensing for gas detection deal largely with greenhouse gases, as suggested by the keywords “carbon dioxide” and “methane emissions”. Moreover, gases affecting air quality are also frequently addressed, such as “nitrogen oxides”, “carbon monoxide” and “sulfur dioxide”. It is noteworthy that the occurrence of the word “emission” poses a challenge when dealing with UAV-based measurements since the attempt to quantify gaseous emissions using drones as mobile platforms is a recent innovation. The earliest publication, according to our search, covering this aspect emerged in 2015 in a study aiming to evaluate the methane leak rate from a natural gas compressor station [29]. The literature on UAV-based emission studies increased starting from 2018: since then, several papers explored technical and theoretical questions related to the quantification of CH_4_ [27,28,39,40,59,60,61,62,63,64], CO_2_ and SO_2_ [65,66] emissions. Further studies have employed UAVs as a device for both air collection and direct measurement at sites that are difficult to access or risky for personnel. Air samples, analysed on the ground with standard techniques, and direct measurements were used to determine the emission factors for various gaseous species (CO, CO_2_, CH_4_, NO, NO_2_ and N_2_O), PM, and VOC deriving from biomass combustion [67,68]. Moreover, the occurrence of the keyword “landfill” related to the thematic group of emission of gases highlights one of the major fields of application of the method. Waste landfills are significant sources of atmospheric methane, the second most important GHG after carbon dioxide [40]. Along with refineries, petrochemical plants and gas production plants, they represent the major sectors of the application of UAV-based sensing for monitoring of leaks of hazardous gases and pollutants. 

The group of keywords connected to air pollution and quality (green group, Figure 4) highlights the thematic fields related to the monitoring objectives (“particulate matter”, “dust”, “aerosol”, “traffic emissions” and “meteorological conditions”), the main scopes and areas of application (“environmental monitoring”, “urban area” and “boundary layer”), the target results (“vertical profile”) and techniques of data analysis (“computational fluid dynamics”). Air quality, as an index of the grade of pollution responsible for adverse effects on human health and well-being, requires constant monitoring in urban areas. The necessity of rapid, low-cost and flexible instruments to regularly check air quality is reflected in the research efforts towards UAV-based monitoring platforms. Particulate matter, according to our search, is the most investigated pollutant in the thematic group of air quality monitoring. The keyword “aerosol” appears less frequently in the analysed literature. Atmospheric aerosol, being referred to as a suspension of solid or liquid particles in air [69], is considered conceptually equivalent to PM. The keyword “dust”, which is defined as solid particles formed by the process of mechanical disruption (Reist, 1993), is also frequently mentioned in the analysed literature. Overall, most papers dealing with field-based measurements using UAVs investigate particulate pollution in urban contexts, as shown by the recurrence of the keyword “traffic emissions”. Few are monitoring experiences in rural contexts, where particulate formation is associated with productive activities such as, e.g., agriculture and farming, and where, nevertheless, the impact on the health of workers or animals is relevant [70]. Finally, several studies explored the 3D measurement potentialities of UAVs to obtain vertical profiles of air pollutants.

The presentation of this map also contributes to the search for publications related to specific fields of sensors embedded in UAVs for the monitoring and evaluation of air pollutants and how authors should organize their keywords for easy viewing.

### 3.5. Trends in UAV-Based Sensors to Monitor Air Pollutant Research

**Figure 5:**
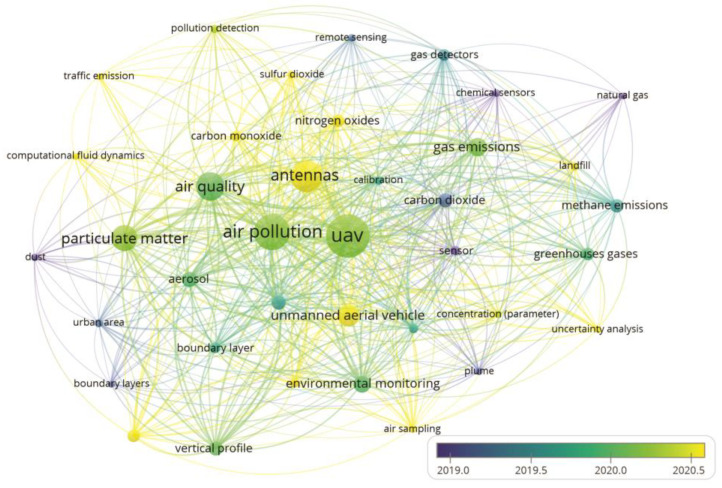
Map based on the co-occurrence of the authors’ keywords and evolution from 2002 to 2022. The colour scale represents the year of keyword predominance.

A network mapping of the keywords most used by the authors in recent years is reported in Figure 5. As expected, the keywords “UAV”, “air pollution” and “air quality” are common keywords in the research, and all other terms co-occur with them.

Figure 5 shows that authors in the past used more specific keywords to monitor air pollutants through sensors embedded in UAVs. Keywords like “greenhouse gases”, “methane emissions”, “chemical sensors” and “gas detector”, among others, are possible. However, over time, authors began to use broader keywords such as the most cited, “UAV”, “air pollution” and “air quality”. As previously noted, the keywords related to sensor technology do not appear in Figure 5, demonstrating a tendency among authors to not include the used sensor technology in the keywords of their studies. Authors use broader, more generic terms to reach a wider audience. The use of broader terms in research addressing sensor-based UAVs in monitoring air pollutants is related to the widespread application of these systems to assessments of different air pollutants in other conditions. The evolution of UAV technology and detection sensors and the worldwide concern for good air quality have boosted applications in the most varied segments, such as pollution assessments on roads, waste sites, wastewater and data mining, among others. Furthermore, the focus of research in the coming years will be the development of UAV-based sensors capable of simultaneously monitoring and evaluating a wide range of atmospheric pollutants.

### 3.6. Study Limitations

The article search process proposed in this study may not include interesting research or may lead to biased or inconclusive results. Many authors need to include the type of sensor used in the titles, keywords or abstracts. In addition, we chose to evaluate only peer-reviewed articles, excluding book chapters, conferences and technical notes, among other research that may provide a new view on the subject. Another limitation, perhaps the most important, concerns the top 10 most important articles. Recent articles can present a paradigm shift; however, as they take time to accumulate citations and gain attention, they still need to be remembered. Furthermore, for a more structured and robust search, more research databases can be included, in addition to Scopus and Web of Science.

## 4. Conclusions

This study’s results allowed for an evaluation of the scientific evolution, research and authorial references on the application of embedded sensors in UAVs for monitoring and evaluating air pollutants. The main countries, journals, scientific organizations, researchers and co-citation networks with greater relevance to the study area were highlighted. The literature, in general, has grown rapidly and attracted enormous attention in the last 5 years, as indicated by the increase in articles after 2017. This increase is related to the worldwide concern about air quality; the creation of stricter laws regarding the atmospheric concentration of pollutants; and the significant progress achieved in the UAV manufacturing sector, combined with advances in sensor technology.

When properly equipped with air quality sensors, UAVs offer a powerful solution to monitor and detect stationary and mobile sources of air pollutant emissions. Thus, it was possible to observe numerous applications of different UAV-based sensors from studies evaluating atmospheric pollutants in urban and industrial areas to studies measuring the concentration of gases in volcanic plumes. Despite the many benefits, the application of embedded sensors in UAVs for monitoring and evaluating air pollutants presents numerous challenges to be solved. These challenges include developing inexpensive and lightweight sensors that reduce payload weight and provide high accuracy to ensure effective monitoring of atmospheric pollutants. Regarding UAVs, developing high-performance batteries can increase the flight time by ensuring the mapping of larger areas. In addition to technical difficulties, policies and laws, which can vary from country to country, make it difficult to standardize the monitoring and evaluation of air pollutants using UAV-based sensors.

The use of UAV-based sensors to monitor and assess air pollutants will continue to grow in the coming years, driven by new sensor technologies and UAVs. In addition, emerging technologies such as 5G networks can provide new monitoring alternatives. When operating on a 5G network, sensor-equipped UAVs benefit from the ultra-high reliability and low-latency connectivity offered by the 5G network. This means they can receive and act quickly on commands from anywhere. 5G helps to reduce the time between sending, receiving and acting on the commands, thus reducing the margin of error that could occur during the flight. On the face of it, a swarm of UAV-based sensors could work together to monitor large areas quickly and safely.

## Figures and Tables

**Table 1:** Top 10 scientific publications on the application of embedded sensors in UAVs for chemical sensing.

R ^1^	Title	Authors	PY ^2^	Journal	NC ^3^
1	Development and Validation of a UAV Based System for Air Pollution Measurements	Villa T.M. et al. [24]	2016	*Sensors*	110
2	Low Power Greenhouse Gas Sensors for Unmanned Aerial Vehicles	Khan A. et al. [38]	2012	*Remote Sensing*	110
3	Development and Integration of a Solar Powered Unmanned Aerial Vehicle and a Wireless Sensor Network to Monitor Greenhouse Gases	Malaver A. et al. [46]	2015	*Sensors*	100
4	Autonomous Gas Detection and Mapping with Unmanned Aerial Vehicles	Rossi and Brunelli [47]	2015	*IEEE Transactions on Instrumentation and Measurement*	98
5	A study of vertical distribution patterns of PM_2.5_ concentrations based on ambient monitoring with unmanned aerial vehicles: A case in Hangzhou, China	Peng Z. et al. [25]	2015	*Atmospheric Environment*	94
6	Towards the Development of a Low Cost Airborne Sensing System to Monitor Dust Particles after Blasting at Open-Pit Mine Sites	Alvarado M. et al. [48]	2015	*Sensors*	90
7	Development of a multicopter-carried whole air sampling apparatus and its applications in environmental studies	Chang C. et al. [49]	2016	*Chemosphere*	66
8	Characterization of a Quadrotor Unmanned Aircraft System for Aerosol-Particle-Concentration Measurements	Brady J.M. et al. [50]	2016	*Environmental Science & Technology*	62
9	Near-Field Characterization of Methane Emission Variability from a Compressor Station Using a Model Aircraft	Nathan B.J. et al. [29]	2015	*Environmental Science & Technology*	56
10	Developing a Modular Unmanned Aerial Vehicle (UAV) Platform for Air Pollution Profiling	Gu Q. et al. [51]	2018	*Sensors*	54

^1^ Rank, ^2^ year of publication, ^3^ number of citations.

**Table 2:** Top 6 sources of publications on the application of onboard sensors in UAVs for the monitoring and evaluation of air pollutants and GHGs from 2012 to 2022.

R	Journal	SJR ^1^	CiteScore ^2^	JCR ^3^	H-i ^4^	ISSN ^5^	ND ^6^	NC ^7^
1	*Sensors*	0.803	6.4	3.847	196	1424-8220	22	446
2	*Atmospheric Environment*	1.383	9.2	5.755	257	1352-2310	10	223
3	*Atmosphere*	0.692	3.7	3.110	46	2073-4433	18	186
4	*Remote Sensing*	1.283	7.4	5.349	144	2072-4292	5	119
5	*Science of The Total Environment*	1.806	14.1	10.753	275	0048-9697	10	84
6	*Atmospheric Measurements Techniques*	1.551	7.4	4.184	97	1867-8548	5	29

^1^ SCImago Journal Rank, ^2^ Scopus index, ^3^ Journal Citation Reports, ^4^ Hirsch index, ^5^ International Standard Serial Number, ^6^ number of documents and ^7^ number of citations.

## Data Availability

No new data were created or analysed in this study. Data sharing is not applicable to this article.
